# Parent Scaffolding of Young Children When Engaged with Mobile Technology

**DOI:** 10.3389/fpsyg.2016.00690

**Published:** 2016-05-10

**Authors:** Eileen Wood, Marjan Petkovski, Domenica De Pasquale, Alexandra Gottardo, Mary Ann Evans, Robert S. Savage

**Affiliations:** ^1^Wilfrid Laurier UniversityWaterloo, ON, Canada; ^2^University of Guelph, GuelphON, Canada; ^3^McGill UniversityMontreal, QC, Canada

**Keywords:** parent–child interactions, shared-media-engagement, children and technology, use of mobile devices, iPad

## Abstract

Shared parent–child experiences while engaged with an iPad^TM^ were examined to determine if and then how parents interact with their children when using mobile digital devices. In total, 104 parent–child dyads participated in an observation session where parent–child interactions using the touchscreen tablet device were video recorded in order to observe first-hand the supports and exchanges between parent and child (age range 46.21–75.9 months). Results indicate that parents provide a great deal of support to their children while interacting with the touchscreen tablet device including verbal, emotional-verbal, physical and emotional-physical supports. The types of support offered did not differ as a function of parent gender or experience with mobile devices (users versus non-users). Overall, parents rated their own experience engaging with the touchscreen tablet and that of their child’s positively. Additional survey measures assessed parents’ perceptions of their child’s technology use and attitudes regarding optimal ages and conditions for introducing and using technology. Most parents indicated a preference for very early introduction to mobile technologies. Implications of these findings are discussed.

## Introduction

In our increasingly technologically advanced world, children are gaining exposure to computer-based technologies earlier and with greater frequency than in previous generations. For example, Carson et al. (2013) found that children 2–4 years of age spend an average of 8.4 min per day engaged with computers. Kabali et al. (2015) found that 60% of parents let their children play with mobile media while running errands, 73% while doing chores around the house, and 65% used mobile media to calm their children. Early interaction with computers is a global phenomenon with the proportions of 3–4-year-olds going online ranging from 25% in the United States to 78% in the Netherlands (Holloway et al., 2013). Concomitant with the ubiquitous presence of computers, is the development of increasingly smaller and yet more sophisticated mobile technologies such as touchscreen tablets and smartphones. These devices permit children access to portable, flexible, and intuitive digital media (e.g., Rideout, 2013). In concert with advances in the development of devices is a proliferation of software programs designed to promote exploration, discovery, play, and development of skills specific to cognitive and social development. It is not surprising then that many parents are turning to computer technology as a means of helping their children to learn and/or entertaining them. Yet, unlike other shared engagement contexts such as shared book reading, or co-viewing television, we know very little about how parents interact with their young children with mobile devices. Given the presence and early introduction of mobile technologies such as touchscreen tablets in the everyday lives of children, it is important to examine and understand how children’s earliest interactions with these mobile computer technologies unfold. The present study investigated parental scaffolding when interacting with their children and mobile devices, specifically iPads^TM^, in an informal setting.

The use of mobile devices may be best facilitated if scaffolding from parents is present. Scaffolding refers to the use of techniques or tools that would allow a child to reach a particular goal that would otherwise be unattainable through unassisted efforts (Wood et al., 1976). Vygotsky (1978) envisioned that guided interactions (e.g., instructional dialog) with an adult could afford a higher level of thinking within the child’s zone of proximal development. In other words, presenting children with tasks that are slightly above their current competence (tasks that are challenging but not overwhelming) while assisting them as needed permits them to achieve and learn beyond what they could do if unaided by an adult (Kohlberg and Mayer, 1972; Hogan and Pressley, 1997; Neumann et al., 2009). Yelland and Masters (2007) identified three different types of scaffolding that occur during interactions with stationary computers: *cognitive, affective, and technical scaffolding*. Cognitive scaffolding involves modeling and asking questions by the parent and facilitates children’s understanding of concepts. Affective scaffolding involves provision of encouragement and feedback. Technical scaffolding refers to effective learning strategies that are built into software design such as immediate feedback and automatic leveling (Grant et al., 2012). The present study expands this understanding of parental scaffolding by examining scaffolding observed in a mobile technology learning context.

Extant research supports the learning potential provided through scaffolding in computer-based learning contexts. For example, the physical introduction to stationary desktop computers was observed to be easier when young children were initiated to the technology while being seated on a parent’s lap with the parent operating the devices (i.e., mouse, keyboard), and later transitioning to the children’s independent use of the computer devices (Calvert et al., 2005). When children acquired the skills needed to control their own activities, they showed greater attentiveness to the tasks and activities than when adults were in control (Calvert et al., 2005). Similarly, in a recent study, pre-test to post-test gains were observed for children’s device specific skills when parents supported their children’s device skills while using stationary desktop computers (Flynn and Richert, 2015). These children also demonstrated cognitive gains for software related content when their parents provided support to enhance understanding of the software content. Thus, parental scaffolding, that encourages children to become independent in controlling their own actions when using computers and provides support in the cognitive tasks at hand, promotes learning.

Interestingly, Flynn and Richert (2015) identified the multiple tasks associated with stationary desktop computers as having the potential to overload children’s working memory and as such interfere with their ability to learn content. The intuitive nature of mobile touch screen tablet devices such as iPads^TM^ reduces the mental and spatial demands required to operate and navigate the device. For example, the touch and swipe actions required for touchscreen tablets remove the complex spatial knowledge required to associate actions with the mouse or keyboard to actions on the screen. These reduced cognitive demands should increase attention to content, and potentially promote greater and more immediate learning with mobile tablet devices than with desktop computers. In addition, the reduced technical demands needed to operate and navigate tablets might also influence the types of scaffolding offered by parents, as attention shifts from ‘learning how to use the technology’ to ‘using the technology to learn.’

Once children acquire the skills to use technology independently it is important that adults monitor and support the ongoing use of the device and software programs to maximize children’s engagement, learning and safety (Espinosa et al., 2006). Promoting these kinds of self-regulatory behaviors in computer-based learning contexts is consistent with expectations in more traditional non-media based learning contexts. Indeed, the ability to be a self-regulated learner is one of the most important factors that separates children who are “successful learners” from children who are “less successful learners” (e.g., Paris and Paris, 2001; Zimmerman, 2002). Research that informs our knowledge about successful learning has been generated primarily from traditional non-media based learning contexts with school-aged children. However, interactions in the home also provide important opportunities for learning both when parents actively and intentionally provide instructional opportunities for their children and, perhaps more frequently, through incidental learning opportunities. Both intentional and incidental learning opportunities allow children to gain exposure to and experience with the precursor skills for self-regulation. Given the increased presence of mobile technologies which permit learning in multiple contexts, sometimes referred to “here and now” learning (Martin and Ertzberger, 2013), it is therefore important, to determine how parents support and encourage foundational skills associated with self-regulated learning in a mobile technology learning context.

Vygotsky (1978) viewed tools of the culture as key mechanisms through which we facilitate the acquisition of higher mental functions. In this regard, the presence of computer-based technological devices and, in particular, recent technologies such as touchscreen tablets, smartphones, and other mobile devices may be viewed as tools of the culture in today’s Western societies. These devices are used to communicate, educate, entertain, and facilitate social interactions and work. As such they serve multiple purposes, some of which directly support and advance higher mental functions. Understanding when parents introduce these cultural tools to their young children and identifying parental supports that facilitate early interactions with these technologies may be key to understanding how these tools are best used to facilitate learning.

Parents own familiarity and skills with technologies also are an important consideration when trying to understand how cultural tools are shared across generations. It could be expected that more knowledgeable and skilled parents might engage their children differently than parents with less knowledge or skills and these differing interactions could alter the learning experience provided to their children. In the present study, parental familiarity with these cultural tools was examined to further understand the impact of familiarity on the exchanges that occur when parents and children are mutually engaged with mobile technology.

Mobile touchscreen tablets are designed in such a way that even very young users can use them easily. Touch-sensitive devices allow for an easier to use and more intuitive interface for children (McManis and Gunnewig, 2012). The size and mobility of the device permits children the flexibility of laying the tablet in their lap, on the floor, or moving with it to any area within their home (their bedroom, their play area, etc.). In addition, the interactive multimedia capabilities of touchscreen tablets can stimulate visual, auditory, tactile, and kinesthetic sensory systems. As well, the response to children’s input is instant, providing immediate feedback (Cooper, 2005; Tahnk, 2011). In effect, these features enable children to quickly learn to use the technology and explore new things, learn new skills, and gain knowledge (McManis and Gunnewig, 2012). Affordances inherent in intuitive devices such as touchscreen tablets provide a context where early introduction is not only likely but expected. Low costs, portability, increasing availability of internet connectedness and a host of available applications make it probable that many parents will be using these mobile devices and that traditional gaps based on socio-economic status may no longer be apparent (e.g., Kabali et al., 2015). However, little research has examined the use of mobile devices with young children especially in the home or by parents (Plowman et al., 2012). Some research studies have examined parent–child interactions with mobile devices such as a LeapPad^TM^ (e.g., Eagle, 2012) and e-books (e.g., Korat and Or, 2010). Where the literature becomes sparse, however, is in examining the interactions between parent and child while using a touchscreen tablet computer. In particular, parents’ scaffolding and support strategies and behaviors, as well as the impact of their familiarity with the mobile device (e.g., novice users as opposed to experienced users) have not been examined.

A great deal of research shows that parents desire to support their children’s learning and seek to provide positive learning environments for their children (Evans and Shaw, 2008; Neumann et al., 2009; Davies, 2011; Eagle, 2012). Parents also view the home and their role as being highly influential in children’s development. For example, over a third of parents rated themselves as being primarily responsible for children’s literacy development (Evans et al., 2004). Evidence in other domains supports the important role parents play in their children’s learning. For example, when parents use more spatially descriptive words (e.g., long, small) during joint activities, their children demonstrate long term gains in spatial word production and competence (Pruden et al., 2011). Learning in the home can be intentional or incidental. The spontaneous and incidental learning that takes place with young children in their home environments is likely to be facilitated by mobile devices as opposed to stationary desktop technology, which requires more skills, space, and planning to use jointly. To fully understand the impact of touchscreen tablets in the context of the family, the present study explored parent–child shared interaction to uncover how parents engage and support their children with these devices.

### The Present Study

Shared parent–child experiences while engaged with an iPad^TM^ were examined to determine if and then how parents interact with their children when using mobile digital devices. Survey measures assessed parents’ perceptions of their child’s technology use and parent’s attitudes regarding optimal ages and conditions for introducing and using technology. A 10-min observational session of mothers and fathers allowed for a first-hand examination of parental scaffolding when using mobile tablet technology with their young children. Given the exploratory nature of the present study, the key research questions involved examining and documenting the different types of supports that parents provided children when engaged interactively using an iPad^TM^. Further, we explored whether parents experienced in the use of mobile devices (users) differed from inexperienced parents (non-users) in the types of supports they offered their child. We also assessed, whether gender differences existed between mothers and fathers and the types of interactions/scaffolds they provided their children. Finally, we examined whether scaffolding behaviors varied according to individual characteristics of the child or parental perceptions of technology.

## Materials and Methods

### Participants

In total, 104 parent–child dyads, 72 mothers (*M*_age_ = 35.40 years, *SD* = 4.81) and 32 fathers (*M*_age_ = 37.10 years, *SD* = 4.85) participated in one interactive touchscreen tablet play session with their 2–6 years old child. There were no significant age differences between mothers and fathers, *t*(102) = 1.86, *p* = 0.07. Most parents indicated some level of higher education: college diploma (13.5%); undergraduate degree (35.6%); Master’s degree (24%); doctorate degree (6.7%); or a post-doctorate (8.7%). A smaller proportion of the sample reported some post-secondary education (6.7%) or a high-school diploma (2.9%). Two participants did not report their education level. Among the parents, 76% self-identified as being familiar with the touchscreen tablet device they were asked to use in the observation session (*n* = 28 males, *n* = 51 females) and 24% were new to the mobile device (*n* = 4 males, *n* = 21 females). Those who self-reported familiarity with touchscreen tablet devices were coded as “users” and those unfamiliar with the devices were considered “non-users” in subsequent analyses. In addition, 20% of non-users (*n* = 5) did not own any computer, laptop, mobile tablet, or iPad^TM^.

Children included 50 girls (*M*_age_ = 46.21 months, *SD* = 13.22, range = 24.3–68.9 months), and 54 boys (*M*_age_ = 44.59 months, *SD* = 14.92, range = 22.8–75.9 months). Overall, there were 32 children under 35 months of age, 31 children aged 36–48 months, 18 children aged 49–60 months, and 20 children over 60 months of age. There was no significant age difference between girls and boys who participated in the study, *t*(102) = -0.58, *p* = 0.56. Participants were recruited from early childhood education and daycare centres in a mid-sized Canadian city. All participants spoke English and used English throughout the observation session. This study was reviewed and approved by a University ethics review board. All participants were treated in accordance with APA ethical standards and were informed of their voluntary participation in all aspects of the study including their choice regarding whether or not to answer any questions on the survey or to participate in the play session.

### Materials

Materials included two surveys (pre- and post-observation) and the observation session.

#### Pre-Observational Survey

The pre-observational survey assessed: demographic information (parent’s gender and age, the child’s gender and age, and the parent’s highest level of education), and parental beliefs regarding the introduction of technology for their child. Timing for the introduction of technology for their children was assessed by asking parents to identify at what age they would introduce technology to their child with answer options that increased in 6-month increments from “Birth” to “After 6 years of age.”

#### Technology

Each parent–child dyad used one iPad^TM^ (Model A1430, version 5.1.1 9B206 operating on iOS 6.1.2). In addition to default applications/software typically available on an iPad^TM^, 12 children’s reading- and math-based applications were downloaded. The 12 applications were chosen based on positive user reviews and ratings. The iPad^TM^ was housed in a spongy jacket called “iGuy^TM^” shaped like a figure with sponge arms and legs. Apart from protection, the jacket enhanced maneuverability by allowing the iPad^TM^ to be held by arms. The case/jacket and also allowed the device to stand independently on its feet when placed on a flat surface.

Video recordings of observation sessions were made using three cameras. Two small cameras were located at either end of the room providing a full, length-wise view of the entire room, and a third small camera provided a view from an elevated position.

#### Post-Observation Survey

The post-observation survey was comprised of 10 questions. Two forced-choice (yes/no) questions assessed whether parents allowed their child to use mobile technologies and if they downloaded programs for their child. For parents who responded “yes” to downloading applications, there was a further prompt for parents to select from 15 possible choices all of the reasons they use for supporting their decision to download applications for their child (see **Table 2** for a list of these rationales).

As a fidelity measure, parents were asked to rate how closely the observation setting reflected typical interactions with their children when engaged with technology using a 5-point Likert-type scale with anchors ranging from “Not at all similar” to “Almost the same.”

Four questions assessed familiarity with, interest in and ease of use of the iPad^TM^. Specifically, parents were asked to identify whether they owned a desktop computer, a tablet (i.e., iPad^TM^, PlayBook^TM^, etc.), both or none of these devices. Parents were also asked, “How familiar were you with the iPad^TM^ we asked you to use^TM^?” (measured on a 5-point Likert-type scale with anchors ranging from “Not at all familiar” to “Completely familiar”), “How interesting did you find the iPad^TM^?” (measured on a 5-point Likert-type scale with anchors ranging from “Not at all interesting” to “Very interesting”), and “With respect to ease of use, how would you rate the iPad^TM^?” (measured on a 5-point Likert-type scale with anchors ranging from “Very difficult to use” to “Very easy to use”).

Parents also rated children’s response to the iPad^TM^ used during the observational setting through three questions including, “How do you think your child responded to the iPad^TM^?” (measured on a 5-point Likert-type scale with anchors ranging from “Did not like it at all” to “Liked it a lot”), “How would you rate your child’s familiarity with the iPad^TM^ we asked you to use?” (measured on a 5-point Likert-type scale with anchors ranging from “Not at all familiar” to “Completely familiar”), and “How would you rate your child’s interest with respect to the iPad^TM^ we asked you to use?” (measured on a 5-point Likert-type scale with anchors ranging from “Uninterested” to “Very interested”).

### Procedures

Recruitment advertisements appealed to mothers and fathers with children between 2 and 6 years of age. Parents were informed that the study “examines how children use technology and parent perceptions about technology use.” Flyers provided an email contact address for interested parents. Parents had the option of completing the pre-observation survey either online or via hard-copy. Some parents completed the survey at home while others completed it on site at the university developmental psychology research lab. Research assistants supervised children for parents who completed the survey on site. The observation session began by welcoming parents into the observation room. The room was organized to reflect a “home” environment with a loveseat, two child-sized tables with two chairs and a large oval alphabet carpet to cover the floor. A brief overview was provided for parents to introduce them to navigation (opening and closing applications, movement within applications, orientation of the device in portrait and landscape mode, volume control buttons, home button to exit applications, and the various menus consisting of default apps and downloaded games), the functions available on the iPad^TM^ and the 12 applications downloaded onto the iPad^TM^. Parent–child dyads were given the iPad^TM^ turned on and set at a comfortable volume level and were free to select from the 12 applications as well as typical applications/functions that appear on most iPads^TM^ (e.g., photo album, camera, music, etc.). Parents were reminded that the purpose of the observations was to better understand how technologies are typically used within the home and parents were encouraged to do what they normally would do with their child. Parent–child dyads were given 10 min to play with the iPad^TM^. Typically two research assistants were involved in each testing session. One research assistant was always present in the observation room to assist with the mobile device or answer questions. This research assistant was seated in a far corner and was instructed to be engaged in other activities (not watching, or making eye contact) except when a parent requested assistance. This research assistant also indicated when the 10 min observation time was completed. Following the observation session, parents were asked to complete the short post-observation survey.

## Results

### Introducing Technology to Children

Parents were asked to indicate the age at which they would consider introducing digital technologies to their children using one of the 12 options encompassing 6 month intervals from birth to 6 years (see **Table 1**). Interestingly 17.5% of parents supported introducing technology in the first year of life. Similarly, the greatest proportion of parents supported introducing technology early with almost a quarter of all parents supporting 1.5–2 years of age (24.3%) and another 19.4% supporting 2–2.5 years of age. Fewer than 10% of parents supported school age or later as the ideal time for introduction. ANOVAs indicated no significant differences in preferred age of introduction between mothers and fathers, *F*(1,101) = 0.01, *p* = 0.91^1^. However, parents with less familiarity (*M* = 6.00, *SD* = 3.48,) with technology (non-users) indicated a much later age for introduction (3–3.5 years of age) in comparison to users (*M* = 4.37, *SD* = 2.48, reflecting ages between the 2–2.5 and 2.5–3 years categories), *F*(1,101) = 6.65, *p* = 0.01, η^2^ = 0.06, and *t*(102) = 2.58, *p* = 0.01.

**Table 1:** Percentage of parents endorsing each age group at which they would introduce technologies to their children.

Age range provided	Gender		Experience		Total
	Male	Female	User	Non-user	
1. Birth – 6 months	6.3%	2.8%	5.1%	0	3.9%
2. Just over 6 months to 1 year	12.5%	13.9%	13.9%	12%	13.6%
3. Just over 1.5–2 years	25%	23.6%	25.3%	20%	24.3%
4. Just over 2–2.5 years	15.6%	20.8%	19%	20%	19.4%
5. Just over 2.5–3 years	9.4%	9.7%	10.1%	8%	9.7%
6. Just over 3–3.5 years	15.6%	5.6%	11.4%	0	8.7%
7. Just over 3.5–4 years	0	4.2%	3.8%	0	2.9%
8. Just over 4–4.5 years	3.1%	5.6%	3.8%	8%	4.9%
9. Just over 4.5–5 years	0	4.2%	0	12%	2.9%
10. Just over 5–5.5 years	6.3%	2.8%	2.5%	8%	3.9%
11. Just over 5.5–6 years	0	0	0	0	0
12. After 6 years of age	6.3%	5.6%	3.8%	12%	5.8%

Parents were asked two questions regarding access to technology. First, over 80% of parents indicated that their children were permitted access to digital technologies. Interestingly, over 94% of these parents allowed access to mobile devices such as the iPad^TM^ used in the present study. Among parents who permitted access to technology, ANOVAs indicated that access to devices such as the iPad^TM^ did not differ as a function of parental gender or technology experience, *F*(1,84) = 1.465, *p* = 0.23 and *F*(1,84) = 0.73, *p* = 0.40, respectively. Further, 80% of these parents indicated that they download applications for their children. Downloading applications did not differ as a function of parental gender or technology experience.

To better understand why parents decide to provide children with access to technology, parents were asked to identify the rationale(s) that supported their decision from a list of 15 possible choices (parents could indicate as many as were appropriate; see **Table 2**). A wide range of rationales were selected with most parents endorsing multiple rationales. Although fun or entertainment was the most highly endorsed rationale (56.7%), several educational goals were also frequently endorsed including promoting development in; problem-solving (53.8%), basic math (53.8%), reading (51%), language (47.1%), and science (26%) as well as building hand-eye coordination (46.2%). The least endorsed rationales included: searching for information (12.5%), learning about history (9.6%) and building social skills (4.8%). Comparisons between mothers and fathers did not yield significant differences among the rationales identified. Chi square analyses were conducted for 11 rationales with sufficient sample size to permit comparisons as a function of technology experience. Given the number of comparisons, a corrected *p* = 0.004 was used. Three comparisons were statistically significant. A greater proportion of parents with technology experience endorsed ‘developing basic skills in math,’ χ^2^ (1, *N* = 104) = 8.85, *p* = 0.003, ‘developing basic skills in reading,’ χ^2^ (1, *N* = 104) = 9.57, *p* = 0.002, and ‘fun/entertainment,’ χ^2^ (1, *N* = 104) = 8.20, *p* = 0.004 as important for introducing technology. In addition there was a strong trend supporting ‘developing basic skills in language,’ χ^2^ (1, *N* = 104) = 7.06, *p* = 0.008 as an additional rationale endorsed by parents with greater technology experience.

**Table 2:** Rationales for introducing children to technology.

	Gender		Experience		Total
	Male	Female	User	Non-user	*N* = 104
Building hand–eye coordination	56.3%	41.7%	53.2%	24%	46.2%
Strengthening reflexes	25%	23.6%	24.1%	24%	24%
Building social skills	9.4%	9.7%	10.1%	8%	9.6%
Building problem-solving skills	56.3%	52.8%	60.8%	32%	53.8%
Developing basic skills in math	56.3%	52.8%	62%	28%	53.8%
Developing basic skills in reading	56.3%	48.6%	9.5%	24%	51%
Developing basic skills in language	53.1%	44.4%	54.4%	24%	47.1%
Developing basic skills in science	28.1%	25%	29.1%	16%	26%
Arts and Crafts	43.8%	26.4%	38%	12%	31.7%
History	6.3%	4.2%	3.8%	8%	4.8%
Searching for information	12.5%	12.5%	12.7%	12%	12.5%
Fun/Entertainment	59.4%	55.6%	64.6%	32%	56.7%
Developing skills for future school success	34.4%	41.7%	44.3%	24%	39.4%
Occupying your child	50%	43.1%	51.9%	24%	45.2%
My child asked for it	18.8%	26.4%	25.3%	20%	24%

### Scaffolding Children during Mobile Technology Play

Three raters worked collaboratively on video files for four observation sessions to identify the types of scaffolding parents offered to their children during the interactive play session with the iPad^TM^. Raters reached consensus in identifying scaffolds. Four types of support were identified: physical, verbal, emotional-verbal, and emotional-physical, plus two additional categories were coded, distractors and off-task behavior. The three raters then independently coded 20% of the video-recorded observation sessions for these categories. Agreement between pairs of raters (raters 1 and 2 and raters 2 and 3) was calculated with high overall inter-rater agreement exceeding 92% for each comparison.

**Physical supports** included holding or adjusting the iPad^TM^ for the child to use, pointing to the screen (both in general and to a specific location), touching (pressing) the screen for the child, and helping the child point to something by a hand-over-hand method.

**Verbal supports** included repetition or clarification of the game instructions, reading aloud something written on the tablet screen (e.g., “so that says, ‘Jack played a ___.”’); providing hints and examples (e.g., “‘A,’ like ‘apple.”’), providing direct/step-by-step instruction (e.g., “now press on the green ‘play’ button.”), asking direct or indirect questions (e.g., “where is the number seven?” and “can you tell me where the triangle is?”), commenting or acknowledging something on the screen (e.g., “look at that, you got three stars”), telling the child to try again (e.g., “try that again.”), and providing the child with corrective statements indicating that they are doing something wrong (e.g., “oops,” “uh-oh”).

**Emotional-verbal supports** consisted of verbal prompts that contained an emotional element including: praise, encouragement (e.g., “you can do it,” “there you go!” “yes, that’s right,”), creating excitement and emotion through sound effects, gasps, and other vocalizations (e.g., “ooh,” “woah!”), and laughing (i.e., creating a positive mood).

**Emotional-physical supports** were identified as physical supports with an emotional element including: touching the child (e.g., scratching or ruﬄing their hair, patting them on the back), physical expressions of praise (e.g., high-five, thumbs-up), shaking the child by the shoulders/their hand when they successfully accomplished something, kissing the child, facial expressions (e.g., smile, frown, grimace, shudder), nodding or shaking their head to indicate approval or disapproval, and cuddling with the child or hugging the child.

Two additional categories (Distracted and Off task) were coded to accommodate momentary off-task behaviors and more sustained off-task behaviors of parents but so few of either category were observed that these categories were not included in any analyses.

A time sampling technique was used to code events in the observation session. Each 10-s interval of the 10-min observation session was sampled for the four types of scaffolding. Interestingly, parents provided a great deal of support to their child in the 10-min session. On average 79 (*SD* = 36.27) verbal supports, 76 (*SD* = 51.83) physical supports, 23 (*SD* = 14.40) emotional-verbal supports, and 6 (*SD* = 9.53) emotional-physical supports were provided during the 10 min sessions (see **Table 3**).

**Table 3:** Mean number of instances for each scaffolding type during 10-min iPad^TM^ observation session.

	Gender		Experience		Total
	Male	Female	User	Non-user	*N* = 102
Scaffolding type	*M (SD)*	*M (SD)*	*M (SD)*	*M (SD)*	*M (SD)*
Physical supports	75.77 (58.82)	76.69 (48.93)	74.53 (53.29)	82.54 (47.32)	76.41 (51.83)
Verbal supports	80.90 (35.32)	78.31 (36.89)	80.19 (37.23)	75.54 (33.43)	79.10 (36.27)
Emotional-verbal supports	22.71 (16.08)	22.76 (13.72)	21.31 (12.54)	27.42 (18.81)	22.75 (14.40)
Emotional-physical supports	3.90 (4.66)	6.61 (10.93)	5.82 (10.53)	5.67 (5.24)	5.78 (9.53)
Distractor	0.48 (1.29)	0.72 (1.42)	0.59 (1.22)	0.83 (1.81)	0.65 (1.38)
Off-task	0.55 (1.06)	0.83 (3.45)	0.41 (1.05)	1.83 (5.69)	0.75 (2.93)

Correlations among these four types of scaffolding were conducted (see **Table 4**). Verbal scaffolding was significantly correlated with emotional-verbal scaffolding and physical scaffolding, *r* = 0.465, *p* < 0.01 and *r* = 0.554, *p* < 0.01, respectively. Emotional-verbal scaffolding also was correlated with emotional-physical scaffolding, *r* = 0.22, *p* < 0.05. No other correlations were significant.

**Table 4:** Correlations among the types of parental scaffolding (i.e., verbal, emotional-verbal, physical, and emotional-physical) provided during the parent–child tablet play session.

	1	2	3	4
1. Verbal scale	–	–	–	–
2. Emotional-verbal scale	0.457**	–	–	–
3. Physical scale	0.556**	0.193	–	–
4. Emotional-physical scale	0.099	0.210*	0.081	–

**p < 0.01 (2-tailed), *p < 0.05 (2-tailed).

Two MANOVAs were conducted to examine whether users and non-users or mothers and father differed in the types of supports they offered their child. Although there were no statistically significant differences between users and non-users for the four scaffolding measures, the emotional-verbal comparison approached significance, *F*(1,102) = 3.14, *p* = 0.08, η^2^ = 0.03. Exploration of this trend suggests that non-users engaged in more emotional-verbal supports (*M* = 27.42, *SD* = 18.81) than users (*M* = 21.58, *SD* = 12.37) in the 10-min iPad^TM^ observation session. There were no significant differences between mothers and fathers on any of these four scaffolding measures, with the largest value being for emotional-physical scaffolding, *F*(1,102) = 1.76, *p* = 0.18.

### Engagement by Children

Scoring of the videos also revealed that children were occasionally off-task or unengaged with the iPad^TM^ activity. Two raters reviewed all observation videos and recorded the total number of times children were off-task as well as the duration of each instance that the child was not engaged. A total time off-task score was calculated by adding all individual off-task periods. Children varied in the number of off-task events with the average number of times off-task being less than two times (range = 0–15 instances; *M* = 1.37, *SD* = 2.80). The average of each instance spent off-task was approximately 13 s (*M* = 12.88, *SD* = 31.11). The duration ranged from 0 to 158.86 s with two outliers of 304.07 and 311.44 s that were greater than 3 standard deviations from the mean. Given these outliers, the previous observational data and subsequent analyses using observational data were re-analyzed without the two outlier children’s scores. No differences in outcomes were noted when these children were added or deleted from the calculations. All data reported in the present results section has these two children’s data deleted from assessments involving the observations. Overall, assessment of children’s off-task behaviors indicated that children spent the vast majority of the time engaged with the technology and if they were not engaged, it was for a short duration.

### Variables Impacting on Scaffolding

To explore whether individual characteristics of parents or children influenced the amount of scaffolding provided, four regression analyses were conducted, one for each of the four types of scaffolding. In all cases the type of scaffolding served as the dependent variable and child age, child gender, parent age, parent gender, and parent experience (user/non-user) served as the predictor variables.

The overall models for verbal scaffolding, *F*(5,101) = 8.09, *p* < 0.001, *R*^2^ = 0.30, and physical scaffolding, *F*(5,101) = 6.07, *p* < 0.001, *R*^2^ = 0.24, were statistically significant. Both verbal and physical scaffolding were predicted by child age, β = -1.44, *r* = -0.53, *t*(101) = -6.2, *p* < 0.001; β = -1.81, *r* = -0.47, *t*(101) = -5.27, *p* < 0.001, respectively, and parent age, β = 1.44, *r* = 0.19, *t*(101) = 2.21, *p* = 0.03; β = 2.57, *r* = 0.23, *t*(101) = 2.61, *p* = 0.01, respectively. As child age increased, the amount of verbal and physical scaffolding parents provided their children decreased. In addition, older parents provided more verbal and physical supports than younger parents.

With respect to the two emotionally based scaffolding supports, neither model was significant.

### Parental Perceptions of the iPad^TM^ Observation Sessions

Parents indicated a moderately high level of interest in the iPad^TM^ device (*M* = 3.85, *SD* = 0.91) and found it relatively easy to use (*M* = 3.85, *SD* = 0.91). When mothers and fathers, and users and non-users, were compared, they did not differ in their ratings of interest or ease of use. A comparison of mothers’ and fathers’ ratings of familiarity with the iPad^TM^ revealed that mothers (*M* = 3.31, *SD* = 1.39) felt less familiar with the device than fathers (*M* = 3.91, *SD* = 1.40), *t*(102) = 2.03, *p* = 0.045. As expected, users (*M* = 3.90, *SD* = 1.27) reported greater familiarity with the iPad^TM^ than non-users (*M* = 2.20, *SD* = 1.04), *t*(102) = 6.08, *p* < 0.001.

Parents were also asked to rate their child’s interest and familiarity with the iPad^TM^ and how much they thought the child liked using it. Four parents did not respond to the interest and liking scales and five parents omitted the familiarity question. Overall, mean interest scores indicated that children were perceived to be very interested in the iPad^TM^ (*M* = 4.46, *SD* = 0.79). Similarly, children were perceived to be very positive about using the iPad^TM^ with mean ratings close to the highest level (*M* = 4.34, *SD* = 0.78) on the 5-point Likert-type scale (5 = “Liked it a lot”). Ratings were also positive, although slightly lower for familiarity with the iPad^TM^ (*M* = 3.21, *SD* = 1.3). Comparisons between mothers and fathers, and users and non-users, revealed no significant differences in ratings. Four regression analyses were conducted to determine if parental perceptions regarding their child’s responsiveness, interest and familiarity with the iPad^TM^ predicted the type of scaffolds they provided (physical, verbal, emotional-physical, emotional-verbal). Three of the four models were significant; Physical [*F*(3,96) = 6.10, *p* < 0.001, *R*^2^ = 0.16], Emotional-Physical [*F*(3,96) = 4.73, *p* < 0.004, *R*^2^ = 0.13], Emotional-Verbal [*F*(3,96) = 7.25, *p* < 0.001, *R*^2^ = 0.19]. The model for Verbal scaffolding approached significance [*F*(3,97) = 2.44, *p* = 0.07, *R*^2^ = 0.07]. In each case higher perceived child familiarity with the iPad^TM^ predicted less scaffolding, Emotional-Physical β = -0.395, *r* = -0.34, *t*(96) = -3.54, *p* < 0.001; Physical β = -0.338, *r* = -0.29, *t*(96) = -3.09, *p* < 0.003; Emotional-Verbal β = -0.380, *r* = 0.33, *t*(96) = -3.53, *p* < 0.001; Verbal β = -0.30, *r* = -0.26, *t*(97) = -2.63, *p* = 0.010 scaffolding. In addition, higher perceived responsiveness of the child predicted more Emotional-Verbal scaffolding β = 0.357, *r* = 0.22, *t*(96) = 2.38, *p* < 0.019.

Finally, parents were asked to report how similar the interactive iPad^TM^ session was to the typical interactions they have at home with their child involving technology. Overall, parents indicated that the session was quite similar to the typical interactions they have with their child involving technology (*M* = 3.62, *SD* = 1.06). No significant differences were found between mothers and fathers and users and non-users.

## Discussion

The two primary goals of the present study were to understand parental perceptions toward introducing mobile technologies to children and to directly observe shared parent–child computer experiences while engaged with an iPad^TM^ to determine if and then how parents use scaffolding with their young children. A growing body of literature from popular media and survey studies suggests that since mobile technologies have become a ubiquitous presence in today’s society, earlier exposure in child populations is becoming more common (e.g., Rideout, 2013; Kabali et al., 2015). The results of the present study confirm parental support for early exposure. Only 9.7% of parents advocated for school-age as the time for introduction. Instead, 43% of parents indicated introduction during infancy (6 months to 2 years) and the majority of parents (61%) supported introduction before 2.5 years of age. There are two important implications that follow from these outcomes. First, early exposure, as noted here, clearly challenges the recommended guidelines regarding screen exposure that is currently advocated by the American Academy of Pediatrics (2001, 2015) that “television and other entertainment media should be avoided for infants and children under age 2. (2015)” The second implication is that parents and family contexts will be the most likely environments in which children gain initial exposure to and use of technologies.

The lack of agreement between what parents believe is good practice regarding the introduction of mobile technologies and what experts in early development indicate as appropriate could signal a potential problem for children developmentally. Specifically, early exposure may limit valuable learning experiences consistent with the deficits identified with passive television viewing (e.g., Napier, 2014) by limiting opportunities to interact with live individuals and limiting active engagement with manipulatives, toys, and the larger environment. Alternatively, it may be the case that developments in the design of software and hardware may have surpassed perceived limitations and could now permit a more active and enriched experience for young children. Although no data are available for infants (aged two and under), a growing body of research supports both learning gains and positive social outcomes when young children use well-designed instructional software (e.g., Willoughby et al., 2009; McKenney and Voogt, 2010; Murray and Olcese, 2011; Tamim et al., 2011; Savage et al., 2013). In addition, the size and flexibility afforded by small mobile technologies such as touchscreen tablets extends children’s learning environments by permitting engagement in multiple contexts rather than the constrained, and perhaps more intentional opportunities associated with desktop computer use.

Interestingly, the age of introduction to technology was influenced by technology experience among parents, with non-users supporting a slightly later introduction age than users. It is not surprising that experienced users would be more likely to introduce the technology to their child as they would be more likely to have opportunities for introduction while using the technology themselves. Even among the non-users, however, the average age of introduction was in the early preschool years (i.e., 3–3.5 years of age). Overall, both the sample in general, and experienced users in particular are likely to invite children to engage with mobile technologies early in development. Thus, understanding the entirety of the parent–child-technology triad becomes a necessity.

The present study indicates that in the best case situation, when being observed, while interacting with their child and technology, parents are engaged. They employ diverse scaffolds to encourage and support their child, and they are positive in their interactions. Parents were observed providing four different types of scaffolding in the interactive iPad^TM^ sessions (i.e., verbal, physical, emotional-verbal, and emotional-physical). Specifically, in the 10-min time span 79 verbal supports and 76 physical supports were offered indicating an average of over 7 of each of these scaffolds per minute. Emotional supports (i.e., emotional-verbal and emotional-physical) were offered less frequently but nonetheless were relatively prominent within the interactions with emotional-verbal supports appearing more frequently than emotional-physical supports. Clearly, parents were actively providing their children with verbal supports to help children understand content, physical supports to aid in manipulating the device and navigating the software, emotional-verbal supports to offer encouragement and praise and emotional-physical supports to acknowledge the child’s successes (e.g., high-five for a job well done).

Neither experience with technology nor gender was predictive of differences in scaffolding. The consistency across genders and users and non-users suggests that features specific to the child or other environmental constraints are responsible for differences in the types of scaffolds parents provide their children. Indeed, with respect to verbal and physical scaffolding, both the child’s age and parental age predicted the amount of scaffolding parents provided their child such that as the age of the child increased, the amount of scaffolding decreased. Importantly, this finding suggests that parents were reducing scaffolding consistent with expected developmental gains in their children’s capabilities, reflecting sensitive scaffolding on the part of parents. Effective scaffolding presumes that supports are tailored to the needs of the learner and this appears to be evident in the present study.

Interestingly, older parents provided more verbal and physical supports than younger parents in the interactive iPad^TM^ session. Existing literature suggests that older parents are more likely to show and feel less stress in their parenting efforts, use better coping strategies and provide more positive reinforcement than younger parents (Auyeung et al., 2011). The current findings suggest these behaviors may translate into more scaffolding in the mobile technology context, perhaps through more graduated scaffolding. Older parents may have persisted longer with verbal and physical supports to fully ensure and reinforce their children’s skill acquisition.

None of the individual characteristic variables that were collected (i.e., child age, child gender, parent age, parent gender, and parental experience) predicted emotional-verbal and emotional-physical scaffolding for the interactive iPad^TM^ session. All sessions were positive. It may be that parents provide emotional supports – both in verbal form such as praise and encouragement and in physical form such as smiles and hugs – naturally as a means to encourage ongoing exploration and engagement. One extension to the current research would be to explore the frequency with which parents shifted across programs and the duration that parents encouraged for particular games, especially those which were either minimally challenging or highly challenging for their child. In the present study, parents could select activities and shift among activities but we did not track specific programs used. Challenges inherent in the software may have an impact on the amount and type of emotional scaffolds required. Further investigation of these emotional supports would be desirable especially as a function of task difficulty where more or fewer supports may be required for effective scaffolding.

Parents demonstrated a desire to support their children’s learning and identified mobile technologies as a platform for achieving educational and entertainment goals. Among those parents (80%) who indicated that they specifically download applications for their children, the majority did so to provide their child with a fun and entertaining experience. This consistency in response indicates that parents believe mobile technologies afford engaging experiences for their children. Several researchers have identified high engagement as a product of children’s software and computers in general (e.g., Willoughby and Wood, 2008). In addition to entertainment, many parents endorsed developing foundational academic skills (i.e., literacy, numeracy) and basic proficiency skills (i.e., hand–eye coordination) as key goals. Neither gender of the parent nor experience with technology discriminated among these rationales. Overall, parents perceive important potential learning outcomes when downloading applications for their young child to use, which is consistent with extant literature associated with quality program design (Grant et al., 2012).

### Parental Attitudes toward the Touchscreen Tablets

Overall, parent’s ratings of the iPad^TM^ technology were generally positive. Perceived interest and ease of use did not differ between mothers and fathers or users and non-users. However, mothers rated themselves less familiar with the technology than fathers. Although gender differences were not expected, they are consistent with some studies indicating that women generally perceive themselves as being less familiar with technologies than men (e.g., Venkatesh et al., 2000) even though actual use or skills may not differ. Consistent with expectations, parents experienced in using mobile technologies reported higher ratings of familiarity.

Parents were also asked to rate how their child responded to the devices used in the present study. Responses were positive. Parents perceived iPad^TM^ play to be engaging for their child. There were no differences between mothers’ and fathers’ ratings or users’ and non-users’ ratings. Parents also rated their child’s familiarity with the device and a comparison of mothers and fathers did not reveal any differences. However, users reported their child as being more familiar with the device than non-users. This may be due to increased exposure to similar mobile devices at home for children of parents who are users. This perceived familiarity among users, however, did not appear to influence the actual scaffolding provided during the observation. It may be that parents who are more familiar with these technologies have generally higher perceptions of familiarity overall but when they engage with their children they scaffold according to the child’s needs rather than perceived skills. With respect to parental ratings of their child’s interest in the iPad^TM^, interest was perceived to be high and there were no differences as a function of technology experience.

### Fidelity within the Study

Several measures were used to ensure that the methods and assumptions involved in the design of the study were evident in the outcomes. Parents’ ratings of the similarity of the observation session to typical interactions they have at home with their child involving technology revealed no differences between mothers and fathers and users and non-users. Importantly, this measure served as a fidelity measure for the observation sessions as parents generally indicated that the sessions reflected their experiences at home rather than a unique experience specific to the lab setting. This was a positive outcome as the study sought to imitate the ‘home’ environment as much as possible. An important next step would be to explicitly examine parent child interactions in the home and perhaps over an extended time frame to more confidently map ‘typical’ and “ideal” behaviors.

### Limitations and Future Directions

One notable limitation in the present study was the small number of non-users relative to users of technology. Recruiting non-users was a challenge. This is perhaps not surprising given the age of the vast majority of the parents in the present study. These parents would fall within the group identified as ‘digital natives’- those who have grown up with technology (Prensky, 2001). Perhaps it was more surprising that 25 non-users were found rather than none. However, the limited number of non-users warrants caution when interpreting the user versus non-user outcomes.

The present study did not include demographic information related to ethnicity and socio-economic status (SES) of participants. However, parental educational level suggests that the current sample was more highly educated than the general population. These factors could potentially play an important role in the way parents interact with their child when using a mobile device, given the increasing use of mobile technologies especially in lower SES groups (Kabali et al., 2015). In addition, future research should consider the relative engagement afforded to mobile technologies versus other important learning opportunities (e.g., shared reading, manipulative play) and the decisions that parents make regarding how they should support their children in these different contexts in order to fully understand how parents allocate support and scaffolding for their children’s learning.

### Conclusion

The present study explored first-hand the nature of the parent–child interactions that take place when children and parents engage in shared-computer activities using a mobile device. The results and implications of this study are important for parents, educators, and childcare providers. Most notably, these parents were very involved and interactive with their child when using the touchscreen tablet. Being an active contributor to children’s learning by providing them with verbal, physical, and emotional support is beneficial, allowing children to engage more actively in the learning tasks through the assistance of a more skilled adult. A second important finding suggests that early introduction to technology is the expectation among parents today, indicating the need to examine very early exposure both in terms of parental support and child learning outcomes. The present study extends the existing literature by examining informal learning contexts between parents and children to see how instruction and support is handled. Gaining an insight into the fundamental behavioral exchanges that occur between parent and child when using mobile technologies may help in understanding how to better support parents and how to support children who have early experiences with technologies. Given positive evidence of the potential for computer assisted instruction in informal learning contexts (Korat and Or, 2010), the present study also provides a foundation for encouraging attention to software development for children, especially very young users. It also suggests to software designers the importance of developing informative and engaging parent portals to support parents who will be scaffolding technology use for their young children.

## Author Contributions

EW and MP participated in each phase of the study from design to the final manuscript. AG contributed significantly to design analyses and writing. DDP contributed significantly to recruitment, data collection and design. RS and MAE contributed to research design and theoretical development.

## Conflict of Interest Statement

The authors declare that the research was conducted in the absence of any commercial or financial relationships that could be construed as a potential conflict of interest.

## Footnotes

1 Separate ANOVA and MANOVA analyses were conducted to examine technology experience and gender differences to accommodate the smaller sample size of non-users.
